# Lactoferrin and Healthy Aging: Gut, Immune, Iron, and Mitochondrial Mechanisms

**DOI:** 10.3390/nu18203319

**Published:** 2026-10-10

**Authors:** Cassandra Evans, Siavash Naddafha, Ross Peterson

**Affiliations:** 1 Dr. Kiran C. Patel College of Osteopathic Medicine Allopathic, Nova Southeastern University, Davie, FL 33328, USA; 2 Helaina Inc., New York, NY 10010, USA; ross@myhelaina.com; 3 School of Medical & Health Sciences, Edith Cowan University, Joondalup, WA 6027, Australia; s.naddafha@ecu.edu.au

**Keywords:** longevity, lactoferrin, glycoprotein, microbiome, immunosenescence

## Abstract

Aging is characterized by progressive dysregulation of interconnected biological systems, including immune function, gut integrity, iron homeostasis, and mitochondrial metabolism, which collectively contribute to inflammaging, oxidative stress, frailty, and age-related disease. Lactoferrin, a multifunctional iron-binding glycoprotein, has emerged as a potential nutritional intervention capable of modulating several hallmarks of biological aging. This narrative review examines current mechanistic and clinical evidence regarding lactoferrin’s role in healthy aging. Evidence from in vitro, preclinical, and human studies suggests that lactoferrin supports gut health, iron regulation, and immune function and may support mitochondrial function and redox balance. Although much of the mechanistic evidence remains preclinical, early human studies demonstrating improvements in inflammatory and iron-related biomarkers support further investigation. Well-designed clinical trials in older adults are needed to determine whether lactoferrin supplementation can meaningfully improve functional outcomes, cardiometabolic health, immune resilience, and markers of biological aging.

## 1. Introduction

Although life expectancy has increased, many older adults experience functional decline driven by dysregulation across interconnected biological systems. Aging reflects not the failure of a single pathway, but the progressive erosion of the cellular defense networks that preserve adaptive capacity and stress resilience [1]. Central to this process are immunosenescence and inflammaging, the progressive weakening of the immune system alongside sustained low-grade inflammation [2,3,4,5,6]. This imbalance increases susceptibility to infection, impaired wound healing, anemia of inflammation, sarcopenia, cardiometabolic instability, neurodegeneration, and frailty. These outcomes reflect overlapping dysfunction across gut integrity, immune regulation, iron homeostasis, increased reactive oxygen species (ROS) production and oxidative stress, and mitochondrial function [3,6]. Although aging is a complex multifactorial process, its trajectory is shaped by modifiable factors such as nutrition, lifestyle, and metabolic health [5,7]. Interventions that target convergent immune, redox, and mitochondrial pathways, therefore, represent promising strategies to support healthy aging.

Lactoferrin (LF) is an iron-binding glycoprotein composed of two homologous lobes, each capable of binding one ferric iron ion [8]. LF is excreted by epithelial cells and is present in other bodily fluids, including saliva, tears, and gastrointestinal fluids. It can also be found in respiratory and digestive tissues [9]. LF varies based on iron saturation, with apo-LF representing the iron-depleted form and holo-LF the iron-saturated form. LF iron saturation varies by source and form. Human and bovine LF differ in amino acid sequence and glycosylation, which may contribute to differences in biological activity [10]. LF is best understood as a multifunctional protein, with pleiotropic biological effects, including prebiotic activity, facilitation of iron absorption, and support of immune function. Clinical studies further indicate that LF promotes the growth of beneficial gut microorganisms and supports intestinal health and immunity [8,11,12,13]. Because LF has been well tolerated in human trials, without a meaningful increase in adverse effects, it has the potential to benefit aging populations.

As the global population of older adults increases, there is a need to identify safe, scalable strategies that preserve functional capacity in later life. Nutritional compounds that modulate immune balance, metabolic homeostasis, and cellular stress resilience offer a practical translational approach. LF is among the best-studied proteins in this context and has demonstrated a favorable safety and tolerability profile in human studies [14,15]. It is generally recognized as safe [16] (GRAS) by the Food and Drug Administration (FDA) and currently used in the dietary supplement and food industries. GRAS status is specific to the source, formulation, and intended conditions of use and should not be interpreted as applying universally to all LF preparations. LF shows promise as a functional food or supplement due to its numerous applications in human health [17,18]. LF has traditionally been studied in the context of host defense, iron regulation, gastrointestinal health, and immune function. Evidence of LF’s effect on healthy aging remains limited. Therefore, the present review focuses on four interconnected domains relevant to aging biology: gut integrity and the microbiome, immune regulation and inflammation, iron homeostasis, and mitochondrial function and redox balance. This review integrates evidence across these domains to evaluate how established LF activities intersect with biological processes implicated in age-related functional decline. Particular attention is given to distinguishing established mechanisms and biomarker effects from emerging mechanistic hypotheses and functional outcomes relevant to healthy aging. A literature search was conducted using PubMed, Google Scholar, and Scopus. Search terms included “lactoferrin,” “aging,” “healthy aging,” “longevity,” “gut microbiome,” “mitochondrial function,” “oxidative stress,” “immunosenescence,” “inflammaging,” “inflammation,” and “iron metabolism.” Reference lists of relevant articles were also reviewed to identify additional studies. Human studies were prioritized when available, with in vitro and animal studies included where clinical evidence was limited or unavailable to provide mechanistic context (Table 1). Evidence is distinguished as direct human evidence in older adults, human evidence from non-older or disease-specific populations, preclinical animal evidence, or in vitro/ex vivo mechanistic evidence. Given the narrative scope of this review, studies were selected based on their relevance to the gut, immune, iron, and mitochondrial pathways.

## 2. Gut Microbiome

### 2.1. Age-Related Changes in the Gut Microbiome

The aging gastrointestinal tract undergoes substantial changes that extend beyond shifts in microbial composition [6]. With advancing age, microbial diversity declines, particularly among short-chain fatty acid (SCFA)-producing taxa such as Faecalibacterium and members of the Lachnospiraceae family [56,57,58]. The decline in SCFA-producing capacity compromises colonocyte energy metabolism, weakens tight junction integrity, and increases intestinal permeability. Additionally, declines in SCFA are associated with weakening of the immune system and increases in inflammation [3,59]. SCFAs support immune function through their effects on cytokine production, innate immune responses, and regulatory T-cell activity. Butyrate has been shown to suppress pro-inflammatory cytokines, including TNF-α and IL-6 [59]. Barrier dysfunction permits translocation of microbial products such as lipopolysaccharide (LPS), amplifying systemic inflammatory signaling and contributing to inflammaging [56]. In parallel, aging is associated with altered mucosal immune responses. Elevated pro-inflammatory cytokines (e.g., IL-6, TNF-α) and impaired secretory IgA dynamics disrupt host–microbe homeostasis [56,57]. These age-related changes are further amplified by polypharmacy, antibiotic exposure, and dietary shifts common in later life, all of which promote dysbiosis and diminish microbial resilience [56]. These changes are hallmarks of biological aging and are believed to actively contribute to frailty and age-associated disease trajectories [56,57,58]. Against this background, LF’s gut-directed effects are especially relevant for maintaining gut homeostasis in later life.

### 2.2. Lactoferrin and the Gut Microbiome

LF plays a multifaceted role in supporting intestinal homeostasis across the lifespan. It promotes epithelial growth, differentiation, and secretory activity, and strengthens barrier integrity by enhancing tight junction protein expression and reducing paracellular permeability [8,19,20]. In vitro studies demonstrate that apo-LF modulates nuclear factor kappa B (NF-κB) and PPAR-γ signaling pathways in intestinal epithelial cells, leading to reduced inflammatory cytokine production and protection against inflammatory injury [19]. LF enhances epithelial barrier function, limiting translocation of microbial products that contribute to systemic inflammaging.

LF also shapes gut microbiome composition. Its high affinity for iron restricts availability to pathogenic bacteria while supporting the growth of beneficial commensals [60]. LF (different types and preparations) exhibits selective antimicrobial activity against organisms such as *E. coli* while promoting probiotic strains including *Lactobacillus acidophilus*, *L. plantarum*, *Pediococcus acidilactici*, and various *Bifidobacterium* species [21,22,23]. Certain probiotic strains express LF-binding proteins, facilitating direct interaction and growth support [61]. LF (bovine) has been shown to increase microbial diversity and enrich beneficial taxa in in vitro models of the aging gut microbiome [35]. In a human clinical study, LF (recombinant human) supplementation maintained microbial diversity while selectively enriching beneficial taxa and modulating short-chain fatty acid production [24]. Although this study does not explore the effects in an elderly population, the findings are relevant; they suggest that LF supports microbiome homeostasis rather than driving broad community restructuring. In a recent preclinical study, available as a preprint, recombinant human LF increased microbial metabolic activity and SCFA production, including butyrate and propionate, while maintaining overall microbial diversity [25]. Additionally, LF (recombinant human) strengthened epithelial barrier function and modulated the response to inflammatory challenge, with increased expression of tight-junction-associated genes and reduced inflammatory signaling. These preclinical findings provide mechanistic support for LF-mediated effects on the gut microbiome and intestinal barrier, processes that may be relevant in the context of age-related dysbiosis, increased intestinal permeability, and chronic low-grade inflammation. In older adults, LF (bovine) supplementation has been associated with increased microbial diversity and enrichment of beneficial taxa, suggesting potential modulation of microbial features associated with age-related dysbiosis [26]. The prebiotic effects of LF (different types and preparations) appear dose-dependent, with lower concentrations supporting commensal growth and higher concentrations exerting antimicrobial effects [23]. These effects may be partly mediated by LF’s high affinity for iron. By sequestering free iron within the intestinal environment, LF can limit iron availability to iron-dependent pathogens, thereby influencing microbial competition and community composition [27]. Microbial metabolites directly influence host mitochondrial bioenergetics and inflammation; thus, shifts toward SCFA-producing taxa may have system-level relevance beyond the gut [6]. It is worth noting that changes in the relative abundance of specific microbial taxa do not directly confer changes or increases in health benefits. Future studies should include functional outcomes to better understand these changes in gut microbiota in response to lactoferrin.

LF supports regulated iron absorption through epithelial LF receptors, whose expression increases during states of intracellular iron deficiency [62]. Iron availability shapes microbial competition within the gut; therefore, LF’s regulation of iron trafficking further contributes to balanced gut microbiome composition and reduced pathogen overgrowth [27]. In addition to microbiota modulation, LF retains iron-binding activity in acidic environments and may support gastric host defense through iron sequestration, interference with epithelial adhesion, and immune-modulatory mechanisms [14,63].

**Figure 1:**
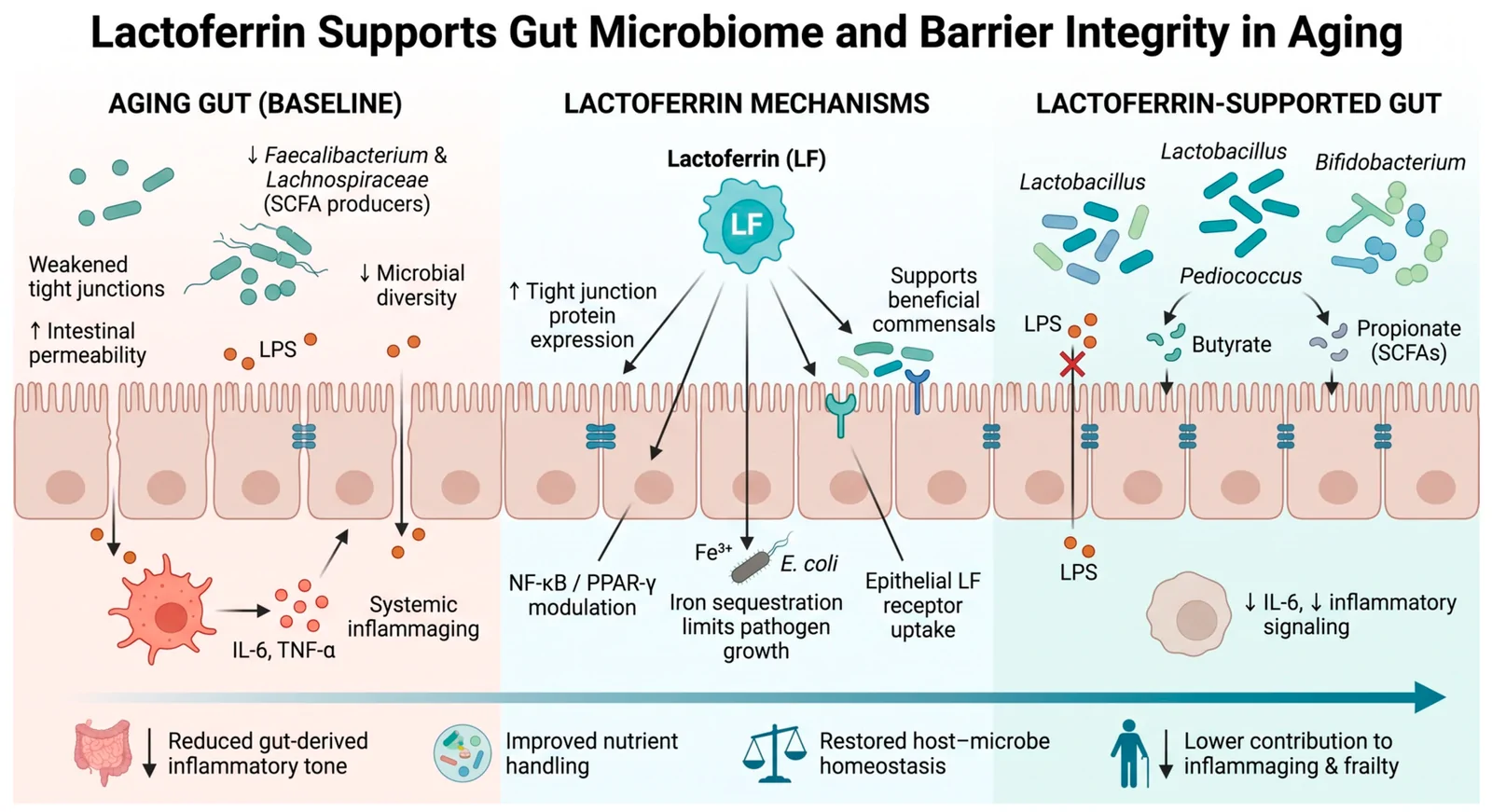
Shows a schematic diagram of potential mechanisms of how lactoferrin supports the gut and barrier integrity in aging. Created in BioRender. Peterson, R. (2026).

Taken together, these findings suggest that LF acts as a regulator of gut barrier integrity, microbial composition, and iron-dependent microbial competition. In aging, where intestinal permeability, dysbiosis, and low-grade inflammation are common, these gut-directed effects may help preserve nutrient absorption, reduce microbially driven inflammation, and support systemic resilience. Given the central role of gut-mediated inflammatory signaling in aging biology, these intestinal effects intersect directly with systemic processes of immunosenescence and inflammaging. Figure 1 summarizes these proposed effects on the gut microbiome and epithelial barrier.

## 3. Immunosenescence and Inflammaging

### 3.1. Mechanisms of Age-Related Immune and Inflammatory Dysregulation

Immunosenescence and inflammaging are closely intertwined processes characterized by impaired adaptive and innate immunity alongside chronic low-grade inflammatory activation [64,65]. Inflammaging is marked by sustained elevations in cytokines such as IL-6 and TNF-α [2]. Senescent cells accumulate and adopt a senescence-associated secretory phenotype that further amplifies inflammatory signaling [66]. Although inflammation is essential for host defense, its chronic dysregulation contributes to tissue dysfunction and increased susceptibility to age-related disease. The balance between pro- and anti-inflammatory cytokines determines whether the inflammatory response remains protective or progresses toward tissue damage and dysfunction. NF-κB serves as a central regulator of inflammatory pathways, and its activity is potentiated by reduced SIRT1 signaling and mTORC1 dysregulation [2]. Declines in autophagy and mitochondrial quality control increase oxidative stress, reinforcing feedback loops between immune dysfunction, metabolic instability, and systemic inflammation [2]. Immunosenescence is characterized by hematopoietic stem cell dysfunction, myeloid-biased differentiation, and reduced adaptive immune output [66]. Natural killer cell cytotoxicity declines, and T cell populations shift toward reduced naïve subsets and expanded highly differentiated phenotypes. B cell diversity decreases, collectively impairing pathogen clearance and vaccine responsiveness [4].

Immunosenescence and inflammaging together create a paradoxical state of diminished immune surveillance coupled with persistent inflammatory activation. Accordingly, regulatory mechanisms that temper inflammatory signaling without suppressing normal immune function are central to healthy aging.

### 3.2. LF, Immunosenescence and Inflammaging

Inflammaging is a key determinant of biological aging and is strongly associated with age-related disease. IL-6 and NF-κB signaling are central to immunosenescence and inflammaging. Thus, LF’s immunomodulatory properties may modulate age-associated inflammatory and immune dysregulation. LF neutralizes LPS and interferes with TLR2, TLR4, and TLR9 signaling via CD14 interaction, thereby limiting downstream NF-κB activation and reducing transcription of pro-inflammatory cytokines including IL-6, TNF-α, and IL-1β [28,29,30]. Clinical and preclinical supplementation studies have demonstrated reductions in systemic inflammatory markers, including IL-6 and C-reactive protein [31,32,33,34,35,36]. By attenuating chronic IL-6/NF-κB activation, LF may reduce inflammatory drivers implicated in frailty, anemia of inflammation, sarcopenia, and broader age-associated functional decline [11]. The magnitude and direction of LF’s effects appear to be dose- and context-dependent, with suppression of excessive cytokine production under inflammatory conditions while preserving physiological immune signaling [30]. LF has been shown to differentially regulate cytokine production depending on host immune status, increasing IL-12 and IFN-γ under certain conditions while suppressing TNF-α and IL-6 in states of chronic inflammation [9,12,27,37].

Inflammaging is reinforced by accumulation of senescent cells and the senescence-associated secretory phenotype (SASP), which perpetuates IL-6–dependent inflammatory loops. LF (bovine and camel) may indirectly mitigate SASP amplification through suppression of NF-κB activation and modulation of inflammatory cytokine production [30,67]. Additionally, LF influences PI3K–Akt and mTORC1 signaling pathways, which may enhance cellular stress resilience and preserve regenerative capacity under inflammatory conditions [38,39]. LF has been shown to activate AMPK and promote autophagic flux via LRP1-dependent signaling, providing a potential mechanism by which LF may influence mTOR-driven inflammatory dysregulation in aging [40]. LF activates AMPK and suppresses mTOR signaling, enhances autophagic flux, reduces oxidative lipid damage (4-HNE), and improves Bcl-2/Bax balance, suggesting that LF may modulate molecular pathways associated with inflammaging [41]. Attenuation of IL-6–NF-κB signaling may extend beyond cytokine suppression, as chronic activation of this pathway contributes to age-associated immune decline. These pathways suggest several interconnected mechanisms through which LF may influence age-associated inflammatory signaling. Modulation of TLR-mediated NF-κB activation may reduce excessive pro-inflammatory cytokine production, while regulation of PI3K–Akt/mTORC1 and AMPK signaling intersects with cellular stress responses and autophagy. Although evidence that LF reverses age-related immune decline remains limited, the aforementioned mechanisms provide a potential link between LF-mediated inflammatory regulation and cellular processes involved in immunosenescence and inflammaging.

Age-associated immune decline increases susceptibility to bacterial and viral infection and reduces vaccine responsiveness. LF contributes to host defense by sequestering iron, limiting microbial proliferation, and interfering with pathogen attachment and entry [8,11,12,32,51,63,68,69]. Beyond its direct antimicrobial actions, LF exerts immunomodulatory effects that influence both innate and adaptive immune compartments.

Neutrophil dysfunction is a hallmark of immunosenescence and is exacerbated by chronic inflammatory exposure. LF supports neutrophil homeostasis by modulating pattern-recognition pathways, including TLR2, TLR4, TLR9, CD14, RAGE, and TREM-1, which regulate chemokine signaling such as CXCL1 and influence neutrophil trafficking across epithelial barriers during tissue injury [29,42,43]. By reducing inflammatory cytokine production, LF may help preserve neutrophil chemotaxis and functional capacity in aging tissues.

LF also influences adaptive immunity. It interacts with CD22 and CD169 to modulate B-cell signaling, potentially counteracting aspects of age-related lymphoid decline [43]. LF also promotes differentiation and activation of T-cell subsets, supporting both cell-mediated and humoral immune responses [32,51,70]. Human supplementation studies demonstrate increases in cytotoxic and helper T-cell activation following short-term dosing [51], and enhanced NK cell activity after longer-term supplementation [70]. In addition, clinical trials report reductions in systemic inflammatory markers, including IL-6 and CRP, alongside modulation of leukocyte activation profiles [31].

**Figure 2:**
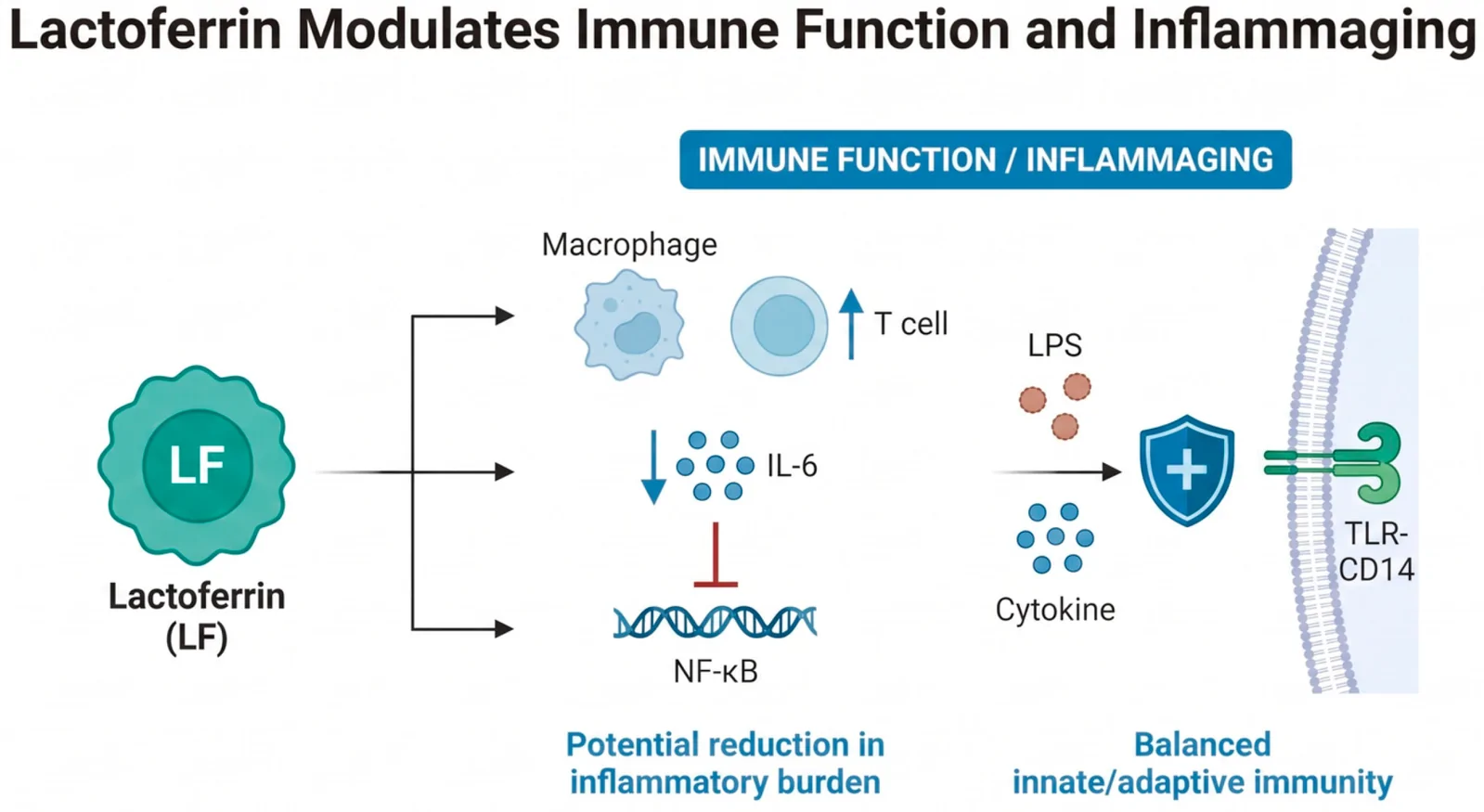
Shows a schematic diagram of potential mechanisms of how lactoferrin acts on the immune system. Created in BioRender. Peterson, R. (2026) https://BioRender.com/jbkrzz3.

Across in vitro, preclinical, and human studies, LF consistently demonstrates immunomodulatory activity characterized by regulation of leukocyte activation, modulation of inflammatory cytokine production, and support of coordinated innate and adaptive immune responses [8,11,12,32,51,68,69]. Importantly, these effects appear dose- and context-dependent, supporting LF’s role as a regulator of immune balance rather than a nonspecific immune stimulant or suppressor. Although human studies support immunomodulatory effects of LF, direct clinical evidence in older adults remains limited, and several of the mechanisms described above are derived from non-older adult populations or preclinical and in vitro models. These findings therefore provide biological and clinical rationale for further study in aging populations rather than direct evidence of reversal of immunosenescence. Figure 2 summarizes these proposed immunomodulatory mechanisms.

## 4. Age-Related Iron Dysregulation

### 4.1. Mechanisms of Age-Related Iron Dysregulation

Iron is essential for cellular metabolism, oxygen transport, DNA synthesis, and mitochondrial energy production [28,71]. Both deficiency and excess compromise cellular function; iron must therefore be tightly regulated. Iron homeostasis becomes increasingly dysregulated during the aging process [72]. Chronic low-grade inflammation increases hepcidin signaling, which suppresses ferroportin activity and drives intracellular iron sequestration [72,73]. As a result, iron may accumulate within tissues despite functional restriction at the systemic level. Ferritin concentrations often rise with age. Ferritin concentrations often rise with age; however, ferritin reflects both body iron stores and inflammatory status. Ferritin is an acute-phase protein, with factors such as age-associated chronic inflammation influencing ferritin levels. This makes interpretation as an isolated marker of iron status challenging. Age-related alterations in cellular and mitochondrial iron handling further contribute to redox imbalance and impaired bioenergetic capacity [72]. Expansion of the labile iron pool enhances reactive oxygen species (ROS) generation and lipid peroxidation, increasing susceptibility to ferroptosis [74]. Senescent cells demonstrate increased intracellular iron deposition, further linking iron dysregulation to hallmarks of aging [72,74]. These changes shift iron from a tightly regulated metabolic cofactor to a contributor of oxidative stress and age-related functional decline.

Disrupted iron homeostasis contributes to functional decline through impaired iron availability and increased oxidative stress. Clinically, anemia in older adults is associated with reduced physical performance, cognitive impairment, frailty, falls, and increased mortality. Among community-dwelling older adults with anemia, approximately one-third have evidence of nutritional deficiencies involving iron, folate, and/or vitamin B12, while another third have renal insufficiency and/or chronic inflammation [75]. Iron deficiency may be absolute, reflecting reduced total body iron, or functional, in which iron sequestration limits its availability for erythropoiesis [72,76]. Conversely, iron excess contributes to cardiometabolic and neurodegenerative diseases, including type 2 diabetes, atherosclerosis, Alzheimer’s disease, and Parkinson’s disease [72,76]. Importantly, both iron overload and iron deficiency carry risk, underscoring the need for balanced iron homeostasis rather than simple iron replacement.

### 4.2. LF and Iron

LF plays a central role in maintaining iron homeostasis under both physiological and inflammatory conditions [8,29,44,45,46]. Structurally, LF binds two ferric iron ions with high affinity and, unlike transferrin, retains iron at the lower pH [16], which is characteristic of inflamed tissues [77]. This enables sequestration of redox-active iron within inflammatory microenvironments, thereby limiting iron-driven ROS formation and lipid peroxidation [8,45]. Through this mechanism, LF directly links iron regulation with oxidative stress control, a central determinant of cellular aging.

**Figure 3:**
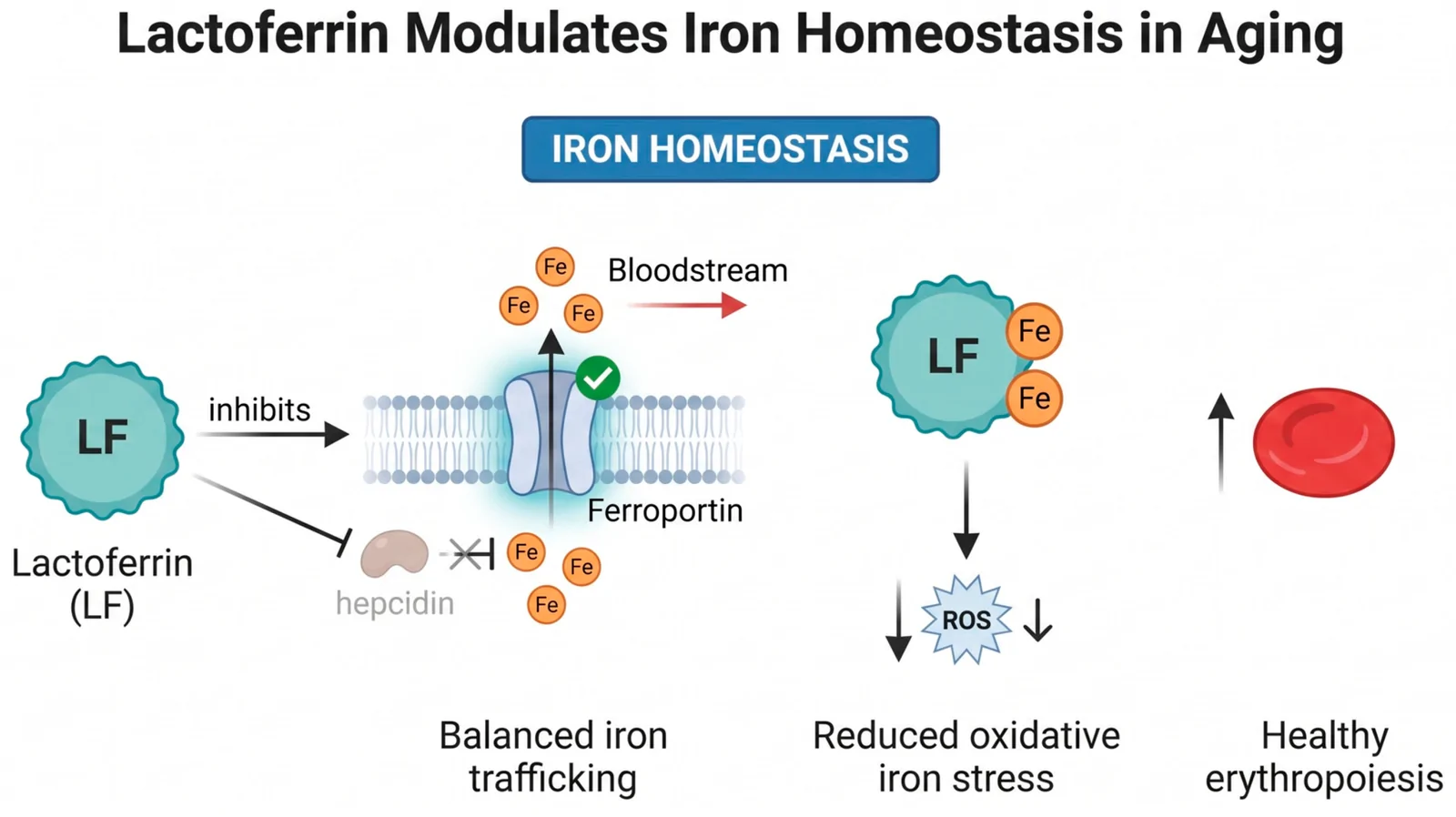
Shows a schematic diagram of proposed mechanisms of how lactoferrin acts on iron homeostasis in aging. Created in BioRender. Peterson, R. (2026) https://BioRender.com/z6v4yac.

LF also modulates key regulators of systemic iron trafficking. As discussed above, inflammatory cytokines such as IL-6 stimulate hepcidin production, which suppresses ferroportin and promotes intracellular iron sequestration. LF (bovine, partially iron-saturated) has been shown to reduce circulating IL-6 levels and influence the hepcidin–ferroportin axis, supporting restoration of iron export and utilization under inflammatory conditions [13]. LF upregulates ferroportin and transferrin receptor expression [47] and activates antioxidant pathways involving Nrf2 [53], further linking iron homeostasis with redox stability and mitochondrial protection. In the gastrointestinal tract, LF binds specific epithelial receptors and enhances iron absorption in states of deficiency while preserving mucosal barrier integrity. Clinical studies demonstrate improvements in hemoglobin and iron parameters following oral LF supplementation, particularly in populations characterized by inflammation-driven iron restriction [13,44,78,79,80,81,82,83,84,85]. Although the consistent improvement in iron biomarkers supports LF’s role as a regulator of iron, most of the clinical evidence has been derived largely from populations other than older adults, limiting direct extrapolation to aging populations. Whether similar effects occur in older adults or translate to functional outcomes relevant to healthy aging remains to be established. By supporting balanced iron distribution while limiting oxidative burden, LF may promote improved erythropoiesis, mitochondrial function, and tissue oxygenation over time. Given the bidirectional risks associated with iron deficiency and iron excess, maintenance of iron homeostasis represents a key component of healthy aging. Human studies examining LF across aging-relevant immune, inflammatory, microbiome, and iron-related outcomes are summarized in Table 2. Figure 3 summarizes LF’s proposed role in iron trafficking and homeostasis.

## 5. Mitochondrial & Redox Aging

### 5.1. Age-Related Mitochondrial Dysfunction

Mitochondrial dysfunction is a central feature of biological aging and contributes to progressive loss of tissue function across cardiovascular, metabolic, musculoskeletal, and neurodegenerative disorders [86,87,88]. Beyond their role in ATP production, mitochondria regulate redox balance, iron handling, inflammatory signaling, and apoptotic pathways [5,87,88]. With advancing age, mitochondrial respiratory efficiency declines and ROS production increases. Increased oxidative stress can damage mitochondrial DNA, lipids, and proteins and interfere with mitochondrial DNA replication and repair [86,87]. Mitochondrial DNA mutations accumulate with age; however, their accumulation does not necessarily indicate a causal role in organismal aging, and their contribution to the aging process is not fully understood. Mitochondrial dysfunction increases the senescent fate of cells [5,88]. As antioxidant defenses and mitophagy become less efficient, dysfunctional mitochondria accumulate, amplifying oxidative and inflammatory stress [86,88]. The latter is due to the release of mitochondrial DNA and metabolites into the cytosol, stimulating inflammasome and activating inflammatory cells [5]. Mitochondrial-derived signals, including mitochondrial DNA, ROS, ATP, and respiratory-chain byproducts, can activate inflammatory pathways, including inflammasome signaling and downstream production of pro-inflammatory cytokines. Changes in iron homeostasis may further contribute to age-related mitochondrial dysfunction. Cellular and mitochondrial iron accumulation has been observed with advancing age, increasing the availability of redox-active iron and potentially contributing to oxidative damage [89,90]. Dysregulated iron homeostasis may intensify this process by increasing mitochondrial ROS production and lipid peroxidation, providing an additional link between impaired mitochondrial iron handling, oxidative stress, and age-related cellular dysfunction [86,89,90].

### 5.2. LF and Redox Balance

Evidence directly linking LF to mitochondrial function in human aging remains limited, with much of the current mechanistic evidence derived from in vitro and preclinical models. LF reduces intracellular ROS generation and attenuates oxidative damage in multiple cell types. In neuronal models of prion-induced toxicity, LF decreased ROS production, preserved mitochondrial membrane potential, prevented cytochrome c release, and inhibited activation of stress kinases and caspase-3, thereby protecting against mitochondrial-mediated apoptosis [48,49]. In endotoxemia models, LF (human) significantly reduced LPS-induced mitochondrial hydrogen peroxide release and limited mitochondrial DNA damage in both cultured cells and animal tissues, including liver, heart, skeletal muscle, and brain [28]. Preservation of mitochondrial DNA integrity may be especially relevant under conditions of inflammatory or age-associated oxidative stress [86,87]. Consistent with these findings, a neonatal inflammatory brain injury model showed that LF (bovine) preserved endogenous antioxidant metabolites and reduced lactate accumulation, indicating improved mitochondrial redox balance under inflammatory stress [50]. Notably, these protective effects exceeded those achieved by iron chelation alone, suggesting that LF’s mitochondrial benefits are not solely attributable to iron sequestration but involve broader regulation of redox and apoptotic signaling pathways.

LF also enhances endogenous antioxidant defense systems. Experimental models demonstrate that LF increases the activity of key antioxidant enzymes, including superoxide dismutase, catalase, and glutathione peroxidase [45,51]. Human supplementation studies have also reported increases in hydrophilic antioxidant capacity, suggesting systemic redox effects [51]. LF supports redox balance at both the mitochondrial and cytosolic levels by limiting iron-catalyzed hydroxyl radical formation while simultaneously promoting antioxidant enzyme capacity.

Iron homeostasis is tightly linked to mitochondrial function, as mitochondria serve as central hubs for iron–sulfur cluster and heme biosynthesis. Dysregulated iron handling promotes mitochondrial ROS amplification and lipid peroxidation, contributing to ferroptotic signaling pathways implicated in aging and cardiometabolic disease [72,91]. LF may indirectly mitigate iron-driven mitochondrial dysfunction and ferroptosis-associated cellular injury through its ability to regulate iron within the body.

LF modulates cell survival signaling networks that intersect with mitochondrial integrity. In osteoblastic and pre-osteoblastic cells, LF reduces apoptosis induced by stress conditions via Wnt5a-dependent activation of cAMP/PKA signaling and stabilization of β-catenin, with partial involvement of PI3K/Akt pathways [52,53]. Although the precise receptor mechanisms remain under investigation, these data suggest that LF influences upstream signaling cascades that regulate mitochondrial permeability, caspase activation, and apoptotic threshold.

LF (bovine) has been shown to directly promote autophagic flux via LRP1-dependent activation of AMPK signaling, enhancing the conversion of LC3-1 (microtubule-associated protein 1A/1B-light chain 3-I) to LC3-II, and optimizing autophagy activity [40]. In primary osteoblasts, LF additionally promotes autophagy through a BCL2–Beclin1-dependent mechanism, reducing BCL2 protein levels, dissociating the BCL2–Beclin1 complex, and enhancing LC3 conversion and autolysosome formation [54]. Given the central role of AMPK–mTOR signaling in autophagy and mitochondrial quality control, these findings support an effect of LF on general autophagic processes. However, these studies did not directly assess mitochondria-specific turnover, and therefore do not establish an effect of LF on mitophagy. In aged mice, LF reduced myocardial lipofuscin accumulation, iron deposition, and oxidative stress [55]. These findings provide additional evidence of LF-mediated effects on oxidative stress and age-associated cellular damage, but do not directly demonstrate enhanced mitophagy. This evidence suggests that LF interacts with mitochondrial redox biology through attenuation of ROS generation, preservation of mitochondrial function, modulation of iron-dependent oxidative chemistry, and regulation of apoptosis-related signaling pathways.

Consistent with these mechanistic findings, preclinical models demonstrate that LF reduces oxidative neuronal injury and preserves brain metabolic integrity under inflammatory stress conditions, supporting a broader neuroprotective role linked to mitochondrial redox regulation [48,51,53]. In macrophage models of atherosclerosis, LF enhances autophagic flux via activation of AMPK and suppression of mTOR signaling, reduces lipid peroxidation (4-HNE), and improves Bcl-2/Bax balance, linking LF to mitochondrial quality control pathways that are central to aging and cardiometabolic disease [41].

**Figure 4:**
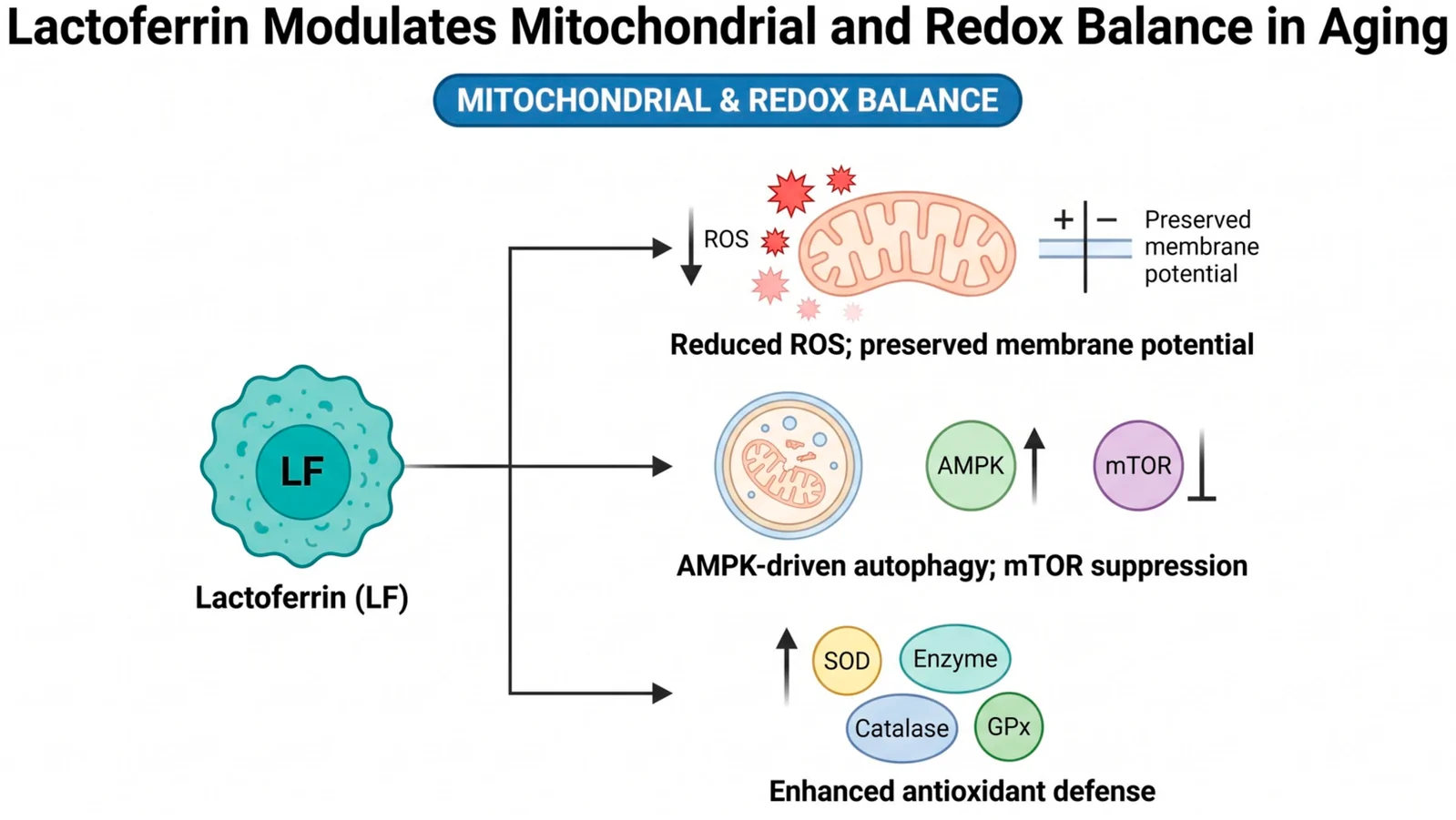
A schematic diagram of the proposed mechanisms of how lactoferrin acts on mitochondrial and redox balance in aging. Created in BioRender. Peterson, R. (2026) https://BioRender.com/cf11v3t.

Although most mechanistic insights derive from cellular and preclinical models (Table 3), they position LF as a potential modulator of mitochondrial stress pathways central to aging biology. Mitochondrial dysfunction, impaired mitophagy, disrupted iron balance, and chronic low-grade inflammation are hallmarks of aging. In the context of age-associated increases in oxidative burden, mitochondrial instability, and chronic inflammatory signaling, LF’s redox-regulatory, iron-modulating, and pro-autophagic properties may contribute to enhanced cellular resilience and maintenance of tissue function. However, whether LF directly modulates mitophagy or mitochondria-specific turnover remains to be established. Given the central role of mitochondrial dysfunction in cardiovascular aging, these redox-stabilizing and mitochondrial-supportive effects may have implications for mitigating age-associated cardiometabolic risk. Preclinical and mechanistic studies supporting the potential effects of LF across the aging-related pathways discussed in this review are summarized in Table 3. Figure 4 summarizes LF’s proposed effects on mitochondrial and redox biology.

## 6. Future Directions

Future studies are needed to determine whether the mechanistic and biomarker effects of LF translate into clinically meaningful benefits in older adults. Trials should prioritize well-characterized older populations and integrate biomarkers of gut barrier function and microbial activity, inflammation and immune function, iron homeostasis, and mitochondrial redox biology with functional outcomes such as physical performance, fatigue, infection burden, cognitive function, and frailty. Careful phenotyping will be particularly important for distinguishing conditions with overlapping biological features, such as iron deficiency and anemia of inflammation, and for determining whether baseline inflammatory or iron status modifies the response to LF. Studies should also include LF-only treatment arms, appropriate dosing durations, longitudinal sampling, and, where feasible, tissue-specific assessments alongside circulating biomarkers. Ultimately, multi-domain trials incorporating both biological and functional endpoints will be necessary to determine whether LF can meaningfully influence the interconnected gut–immune–iron–mitochondrial pathways implicated in healthy aging (Table 4). Lastly, some of the emerging evidence discussed in this review, including studies evaluating recombinant human LF, is company-associated and has not yet been independently replicated. In addition, some findings are derived from preprint or non-peer-reviewed evidence. These findings should therefore be interpreted as preliminary and distinguished from independently replicated, peer-reviewed evidence.

## 7. Conclusions

Aging is marked by progressive disruption of gut integrity, immune coordination, iron homeostasis, and mitochondrial function. These interconnected systems contribute to chronic low-grade inflammation, oxidative stress, and functional decline. Age-related alterations in microbial composition, barrier integrity, immune function, iron handling, and mitochondrial stability may form a self-reinforcing network that amplifies systemic inflammation and diminishes physiological resilience.

LF demonstrates regulatory activity that aligns with key drivers of biological aging: preservation of epithelial barrier function, modulation of microbial composition, regulation of iron trafficking, attenuation of NF-κB–IL-6 signaling, enhancement of antioxidant defenses, and support of autophagic and mitochondrial quality control pathways. Although much of the mechanistic evidence remains preclinical, emerging human studies suggest favorable effects on inflammatory biomarkers, immune activation profiles, and iron parameters. However, whether these mechanistic and biomarker effects translate to meaningful functional outcomes in older adults remains to be established. Given the multidimensional nature of aging biology, interventions that restore systemic homeostasis across gut–immune–iron–mitochondrial axes may offer greater translational potential than single-pathway strategies. The multifaceted effects of lactoferrin suggest it may benefit the aging population and warrants further investigation. Important knowledge gaps remain regarding the optimal LF dose, duration and formulation in aging populations, as well as the identification of older adults most likely to benefit from supplementation. Well-designed clinical trials in older adults with commercially available LF, particularly those assessing functional outcomes such as physical performance, cardiometabolic markers, immune resilience, and measures of biological aging, are needed to determine whether LF supplementation can serve as an adjunct to healthy aging strategies.

## Figures and Tables

**Table 1:** Integrative framework linking biological aging disturbances, candidate lactoferrin mechanisms, and downstream healthy aging relevance.

Aging-Related Domain	Age-Related Disturbance	Proposed Lactoferrin Mechanisms	Potential Relevance to Healthy Aging	References
Gut microbiome and barrier	Loss of microbial diversity; depletion of SCFA-producing taxa; weakened tight junctions; increased intestinal permeability and microbial translocation.	Promotes epithelial growth and barrier integrity; modulates NF-kB/PPAR signaling; restricts iron to pathogens while supporting beneficial commensals; may enrich probiotic taxa.	Reduced gut-derived inflammatory tone; better nutrient handling; improved host–microbe homeostasis; lower contribution to inflammaging and frailty.	[8,19,20,21,22,23,24,25,26,27]
Immunosenescence and inflammaging	Reduced adaptive immune reserve with chronic low-grade inflammation; impaired pathogen defense; diminished vaccine responsiveness.	Neutralizes LPS; dampens TLR-CD14 signaling; reduces NF-kB-linked cytokine transcription; modulates innate and adaptive immune activation in a context-dependent manner.	Potential reduction in inflammatory burden, infection susceptibility, frailty-associated immune dysfunction, and broader age-related immune decline.	[9,12,27,28,29,30,31,32,33,34,35,36,37,38,39,40,41,42,43]
Iron dysregulation	Inflammation-driven hepcidin elevation; ferroportin suppression; intracellular iron sequestration; ferritin rise; labile iron expansion and redox stress.	High-affinity iron binding even in acidic/inflammatory settings; may normalize hepcidin-ferroportin signaling; supports iron redistribution and deficiency correction while limiting iron-driven oxidative injury.	Healthier erythropoiesis, oxygen transport, mitochondrial support, and lower risk from both iron restriction and iron excess.	[8,13,29,44,45,46,47]
Mitochondrial and redox aging	Respiratory inefficiency; higher ROS production; mitochondrial DNA (mtDNA) damage; impaired mitophagy/autophagy; apoptosis susceptibility.	Reduces intracellular ROS; preserves mitochondrial membrane potential; supports (AMP-activated protein kinase) AMPK-dependent autophagy; suppresses (mechanistic target of rapamycin) mTOR-linked stress pathways; enhances antioxidant defenses.	Greater cellular resilience, preserved bioenergetics, and mitigation of oxidative–inflammatory feedback loops that accelerate cardiometabolic and neurodegenerative aging.	[28,40,45,48,49,50,51,52,53,54,55]

**Table 2:** Human evidence summary with direct or indirect relevance to healthy aging.

Reference	Population	Design/Intervention	Main Findings	Aging-Related Interpretation
Berthon et al. [31]	Healthy older adults	RCT; oral bovine lactoferrin supplementation	Reduced systemic inflammation, enhanced antiviral responses, and modulated immune-cell profiles.	Most direct support for healthy-aging relevance through immune and inflammatory endpoints.
Kawakami et al. [70]	Elderly individuals	Randomized double-blind placebo-controlled trial; enteric-coated bovine lactoferrin	Improved immune-function markers after supplementation.	Supports immunosenescence-focused application, although endpoint breadth remains limited.
Konstanti et al. [26]	Healthy elderly women	Multicomponent nutritional intervention including bovine lactoferrin	Gut microbiota composition shifted toward a more favorable profile.	Aging-relevant, but attribution to lactoferrin alone is limited.
Bharadwaj et al. [33]	Postmenopausal women	Supplementation with milk ribonuclease-enriched lactoferrin	Inflammatory responses improved during intervention.	Suggests benefit in a hormonally and immunologically relevant aging population.
Mulder et al. [51]	Healthy adult males	Human supplementation study	Improved immune and antioxidant status.	Supports biological plausibility, but not specifically in older adults.
Vitetta et al. [69]	Adults with common-cold outcomes	Double-blind randomized study with bovine lactoferrin/whey Ig-rich fraction	Clinical efficacy against common-cold burden was reported.	Supports host-defense relevance, though mixed formulation complicates attribution.
Oda et al. [68]	Japanese adults in summer	Randomized double-blind placebo-controlled trial with bovine lactoferrin	Reduced infectious-disease burden/improved infection-related outcomes.	Human support for immune resilience; aging specificity is limited.
Rosa et al. [35]	Individuals with iron/inflammatory homeostasis disorders	Oral bovine lactoferrin; administration-mode comparison	Improved iron and inflammatory homeostasis, with efficacy influenced by administration strategy.	Important translational signal for inflammatory iron restriction relevant to older adults.
Zhao et al. [85] and Christofi et al. [44]	Clinical trial aggregates	Meta-analysis evidence in low-hemoglobin/iron-deficiency settings	Lactoferrin improved iron-related outcomes with favorable tolerability versus conventional iron strategies.	Strong indirect support for iron-regulatory utility, though not age-specific.

**Table 3:** Preclinical and mechanistic studies that strengthen biological plausibility for the lactoferrin-healthy aging hypothesis.

Reference	Experimental Context	Principal Mechanistic Finding	Dominant Pathway
Wu et al. [19]	Bovine lactoferrin effect on LPS-induced intestinal inflammatory injury	Iron-free lactoferrin reduced inflammatory injury via NF-kB/PPAR modulation.	Barrier–inflammatory crosstalk
Aizawa et al. [40]	Bovine lactoferrin effect on cellular autophagy model	Lactoferrin activated AMPK through LRP1 and increased autophagic flux.	AMPK–autophagy
Xia et al. [41]	Lactoferrin effect on macrophage/atherosclerosis model	Enhanced autophagy, suppressed mTOR signaling, lowered 4-HNE, and improved Bcl-2/Bax balance.	Autophagy–redox–survival signaling
Park et al. [39]	Bovine lactoferrin effect on Human mesenchymal stem cells under oxidative stress	Protected against senescence and apoptosis.	Stress resilience/PI3K-Akt-related survival biology
Kruzel et al. [28]	Human milk lactoferrin effect on cultured cells and animal endotoxemia	Reduced LPS-induced mitochondrial hydrogen peroxide release and mtDNA damage.	Mitochondrial redox protection
Park et al. [49]	Bovine lactoferrin effect on prion-induced neuronal toxicity	Preserved mitochondrial membrane potential and reduced apoptosis signaling.	Mitochondrial apoptosis control
de la Rosa et al. [42]	Bovine lactoferrin effect on immune recruitment/APC activation	Acted as an alarmin and promoted APC recruitment and antigen-specific responses.	Innate–adaptive immune coordination
Mohamed and Schaalan [29]	Camel lactoferrin effect on Type 2 diabetic pediatric model	Affected PPAR-gamma, SIRT1, and TLR4 downstream signaling.	Metabolic-inflammation signaling

**Table 4:** Future Study Considerations.

Age-Related Domains	Recommended Endpoints	Design Priorities
Gut barrier and microbiome	**Biomarkers:** Microbiome diversity; SCFA profile; stool taxa; zonulin/permeability markers; circulating LPS-related markers. **Other Endpoints:** GI symptom burden; nutritional tolerance; infection burden; frailty-linked inflammatory score.	Need lactoferrin-only arms, older cohorts, and stool plus blood sampling across time.
Inflammaging	**Biomarkers:** IL-6; TNF-alpha; CRP; leukocyte phenotypes; NK-cell activity; vaccine-response markers. **Other Endpoints:** Respiratory infection incidence; vaccine responsiveness; days ill; recovery trajectories.	Use standardized baseline inflammatory stratification and adequate dosing duration.
Iron homeostasis	**Biomarkers:** Hemoglobin; ferritin; hepcidin; transferrin saturation; soluble transferrin receptor; ferroportin-related readouts where possible. **Other Endpoints:** Fatigue; physical performance; anemia prevalence; exercise tolerance; quality of life.	Differentiate iron deficiency from anemia of inflammation and include tolerability comparisons with ferrous salts.
Mitochondrial/redox biology	**Biomarkers:** Oxidative stress markers; mtDNA damage; antioxidant enzyme activity; lipid peroxidation markers. **Other Endpoints:** Walking capacity; muscle endurance; cognition-related metrics; cardiometabolic risk markers.	Pair circulating markers with functional phenotypes and, where feasible, tissue-specific substudies.
Integrated healthy-aging trials	**Biomarkers:** Composite biological-age signatures; inflammatory, iron, and mitochondrial panels. **Other Endpoints:** Frailty index; sarcopenia metrics; falls; hospitalizations; physical function batteries.	Multi-domain endpoint strategy with pragmatic real-world dosing and safety follow-up.

## Data Availability

No new data were created or analyzed in this study. Data sharing is not applicable to this article.

## References

[1] Zhong D., Sun Y., Zhao L., Hu Z., Li G., Li H., Xie Z. (2025). Interplay between aging and metabolic diseases: From molecular mechanisms to therapeutic horizons. Med. Rev..

[2] Li X., Li C., Zhang W., Wang Y., Qian P., Huang H. (2023). Inflammation and aging: Signaling pathways and intervention therapies. Signal Transduct. Target. Ther..

[3] Nguyen T.Q.T., Cho K.A. (2025). Targeting immunosenescence and inflammaging: Advancing longevity research. Exp. Mol. Med..

[4] Xia S., Zhang X., Zheng S., Khanabdali R., Kalionis B., Wu J., Wan W., Tai X. (2016). An Update on Inflamm-Aging: Mechanisms, Prevention, and Treatment. J. Immunol. Res..

[5] Xu X., Pang Y., Fan X. (2025). Mitochondria in oxidative stress, inflammation and aging: From mechanisms to therapeutic advances. Signal Transduct. Target. Ther..

[6] Zachos K.A., Gamboa J.A., Dewji A.S., Lee J., Brijbassi S., Andreazza A.C. (2024). The interplay between mitochondria, the gut microbiome and metabolites and their therapeutic potential in primary mitochondrial disease. Front Pharmacol..

[7] Gao L., Liu X., Luo X., Lou X., Li P., Li X., Liu X. (2023). Antiaging effects of dietary supplements and natural products. Front Pharmacol..

[8] Kowalczyk P., Kaczyńska K., Kleczkowska P., Bukowska-Ośko I., Kramkowski K., Sulejczak D. (2022). The Lactoferrin Phenomenon-A Miracle Molecule. Molecules.

[9] Hao L., Shan Q., Wei J., Ma F., Sun P. (2019). Lactoferrin: Major Physiological Functions and Applications. Curr. Protein Pept. Sci..

[10] Lu X. (2024). Characterization of recombinant human lactoferrin expressed in Komagataella phaffii. Analyst.

[11] Actor J.K., Hwang S.A., Kruzel M.L. (2009). Lactoferrin as a natural immune modulator. Curr. Pharm. Des..

[12] Embleton N.D., Berrington J.E., McGuire W., Stewart C.J., Cummings S.P. (2013). Lactoferrin: Antimicrobial activity and therapeutic potential. Semin Fetal Neonatal Med..

[13] Mahmoud R.M.A., Mohammed A. (2023). Lactoferrin: A Promising New Player in Treatment of Iron Deficiency Anemia in Patients on Regular Hemodialysis: A Randomized Controlled Trial. Saudi J. Kidney Dis. Transplant..

[14] Di Mario F., Aragona G., Bó N.D., Cavallaro L., Marcon V., Olivieri P., Benedetti E., Orzès N., Marin R., Tafner G. (2006). Bovine lactoferrin for Helicobacter pylori eradication: An open, randomized, multicentre study. Aliment Pharmacol. Ther..

[15] Vishwanath-Deutsch R., Dallas D.C., Besada-Lombana P., Katz L., Conze D., Kruger C., Clark A.J., Peterson R., Malinczak C.-A. (2024). A review of the safety evidence on recombinant human lactoferrin for use as a food ingredient. Food Chem. Toxicol..

[16] Cao X., Ren Y., Lu Q., Wang K., Wu Y., Wang Y., Zhang Y., Cui X.-S., Yang Z., Chen Z. (2023). Lactoferrin: A glycoprotein that plays an active role in human health. Front. Nutr..

[17] Giansanti F., Panella G., Leboffe L., Antonini G. (2016). Lactoferrin from Milk: Nutraceutical and Pharmacological Properties. Pharmaceuticals.

[18] Jańczuk A., Brodziak A., Czernecki T., Król J. (2022). Lactoferrin-The Health-Promoting Properties and Contemporary Application with Genetic Aspects. Foods.

[19] Wu H., Fan L., Gao Y., Wang J., Zheng N. (2022). The Protective Effects of Iron Free Lactoferrin on Lipopolysaccharide-Induced Intestinal Inflammatory Injury via Modulating the NF-κB/PPAR Signaling Pathway. Foods.

[20] Zhao C., Chen N., Ashaolu T.J. (2023). Prebiotic and modulatory evidence of lactoferrin on gut health and function. J. Funct. Foods.

[21] Grzywacz K., Butcher J., Romain G., Li J., Stintzi A. (2019). The impact of probiotics and lactoferrin supplementation on piglet gastrointestinal microbial communities. Biometals.

[22] Liu N., Feng G., Zhang X., Hu Q., Sun S., Sun J., Sun Y., Wang R., Zhang Y., Wang P. (2021). The Functional Role of Lactoferrin in Intestine Mucosal Immune System and Inflammatory Bowel Disease. Front Nutr..

[23] Liu Z.S., Chen P.W. (2023). Featured Prebiotic Agent: The Roles and Mechanisms of Direct and Indirect Prebiotic Activities of Lactoferrin and Its Application in Disease Control. Nutrients.

[24] Peterson R., van der Made J., Donovan S.M., Wang M., Dilger R.N., Kaplan N., Clark A.J. (2026). Effects of Human Lactoferrin (effera^®^) at Two Doses versus Bovine Lactoferrin on the Adult Gut Microbiome and Fecal Short-Chain Fatty Acids: A Randomized, Double-Blind Trial. J. Diet. Suppl..

[25] Kaplan N., Gadde R., Peterson R., Van den Abbeele P., Clark A. (2026). Microbiome-mediated Biotransformation of Human Lactoferrin (effera) Enhances Epithelial Barrier Function in an Ex Vivo Adult Model. bioRxiv.

[26] Konstanti P., van Splunter M., Brink E.v.D., Belzer C., Nauta A., van Neerven R.J.J., Smidt H. (2022). The Effect of Nutritional Intervention with Lactoferrin, Galactooligosacharides and Vitamin D on the Gut Microbiota Composition of Healthy Elderly Women. Nutrients.

[27] Lee B., Moon K.M., Kim C.Y. (2018). Tight Junction in the Intestinal Epithelium: Its Association with Diseases and Regulation by Phytochemicals. J. Immunol. Res..

[28] Kruzel M.L., Actor J.K., Radak Z., Bacsi A., Saavedra-Molina A., Boldogh I. (2010). Lactoferrin decreases LPS-induced mitochondrial dysfunction in cultured cells and in animal endotoxemia model. Innate Immun..

[29] Mohamed W.A., Schaalan M.F. (2018). Antidiabetic efficacy of lactoferrin in type 2 diabetic pediatrics; controlling impact on PPAR-γ, SIRT-1, and TLR4 downstream signaling pathway. Diabetol. Metab. Syndr..

[30] Yami H.A., Tahmoorespur M., Javadmanesh A., Tazarghi A., Sekhavati M.H. (2023). The immunomodulatory effects of lactoferrin and its derived peptides on NF-κB signaling pathway: A systematic review and meta-analysis. Immun. Inflamm. Dis..

[31] Berthon B.S., Williams E.J., Williams L.M., Budden K.F., Hiles S.A., Bartlett N.W., Wood L.G. (2026). Oral lactoferrin reduces systemic inflammation, enhances anti-viral responses and modulates immune cell profiles: A randomised controlled trial in healthy, older adults. Br. J. Nutr..

[32] Berthon B.S., Williams L.M., Williams E.J., Wood L.G. (2022). Effect of Lactoferrin Supplementation on Inflammation, Immune Function, and Prevention of Respiratory Tract Infections in Humans: A Systematic Review and Meta-analysis. Adv. Nutr..

[33] Bharadwaj S., Naidu T.A.G., Betageri G.V., Prasadarao N.V., Naidu A.S. (2010). Inflammatory responses improve with milk ribonuclease-enriched lactoferrin supplementation in postmenopausal women. Inflamm. Res..

[34] Martínez-García J.J., Canizalez-Roman A., Angulo-Zamudio U.A., Velazquez-Roman J., Flores-Villaseñor H., Valdez-Flores M.A., Rios-Burgueño E., Moran-Portela D., León-Sicairos N. (2022). Lactoferrin and Metoprolol Supplementation Increase Mouse Survival in an Experimental LPS-Induced Sepsis Model. Int. J. Pept. Res. Ther..

[35] Rosa L., Lepanto M.S., Cutone A., Siciliano R.A., Paesano R., Costi R., Musci G., Valenti P. (2020). Influence of oral administration mode on the efficacy of commercial bovine Lactoferrin against iron and inflammatory homeostasis disorders. Biometals.

[36] Maritati M., Comar M., Zanotta N., Seraceni S., Trentini A., Corazza F., Vesce F., Contini C. (2017). Influence of vaginal lactoferrin administration on amniotic fluid cytokines and its role against inflammatory complications of pregnancy. J. Inflamm..

[37] Hung C.M., Yeh C.-C., Chen H.-L., Lai C.-W., Kuo M.-F., Yeh M.-H., Lin W., Tu M.-Y., Cheng H.-C., Chen C.-M. (2010). Porcine lactoferrin administration enhances peripheral lymphocyte proliferation and assists infectious bursal disease vaccination in native chickens. Vaccine.

[38] Bournazou I., Pound J.D., Duffin R., Bournazos S., Melville L.A., Brown S.B., Rossi A.G., Gregory C.D. (2009). Apoptotic human cells inhibit migration of granulocytes via release of lactoferrin. J. Clin. Investig..

[39] Park S.Y., Jeong A.-J., Kim G.-Y., Jo A., Lee J.E., Leem S.-H., Yoon J.-H., Ye S.K., Chung J.W. (2017). Lactoferrin Protects Human Mesenchymal Stem Cells from Oxidative Stress-Induced Senescence and Apoptosis. J. Microbiol. Biotechnol..

[40] Aizawa S., Hoki M., Yamamuro Y. (2017). Lactoferrin promotes autophagy via AMP-activated protein kinase activation through low-density lipoprotein receptor-related protein 1. Biochem. Biophys. Res. Commun..

[41] Xia B., Liang J., Lu Y., Ding J., Peng J., Li F., Dai J., Liu Y., Wang J., Wan C. (2025). Lactoferrin influences atherosclerotic progression by modulating macrophagic AMPK/mTOR signaling-dependent autophagy. Sci. Rep..

[42] de la Rosa G., Yang D., Tewary P., Varadhachary A., Oppenheim J.J. (2008). Lactoferrin acts as an alarmin to promote the recruitment and activation of APCs and antigen-specific immune responses. J. Immunol..

[43] Kim J.W., Lee J.S., Choi Y.J., Kim C. (2025). The Multifaceted Functions of Lactoferrin in Antimicrobial Defense and Inflammation. Biomolecules.

[44] Christofi M.D., Giannakou K., Mpouzika M., Merkouris A., Stylianide M.V., Charalambous A. (2024). The effectiveness of oral bovine lactoferrin compared to iron supplementation in patients with a low hemoglobin profile: A systematic review and meta-analysis of randomized clinical trials. BMC Nutr..

[45] Li B., Zhang B., Liu X., Zheng Y., Han K., Liu H., Wu C., Li J., Fan S., Peng W. (2022). The effect of lactoferrin in aging: Role and potential. Food Funct..

[46] McCarthy E.K., O’Callaghan T.F. (2024). Bovine lactoferrin and its potential use as a functional ingredient for tackling the global challenge of iron deficiency. Curr. Opin. Food Sci..

[47] Bolesławska I., Bolesławska-Król N., Jakubowski K., Przysławski J., Drzymała-Czyż S. (2025). Lactoferrin—A Regulator of Iron Homeostasis and Its Implications in Cancer. Molecules.

[48] Al-Sharif I.A. (2023). Protective effect of lactoferrin administration against brain tissue damage in diabetic rats. J. Umm Al-Qura Univ. Appl. Sci..

[49] Park Y.G., Jeong J.-K., Lee J.-H., Lee Y.-J., Seol J.-W., Kim S.-J., Hur T.-Y., Jung Y.-H., Kang S.-J., Park S.-Y. (2013). Lactoferrin protects against prion protein-induced cell death in neuronal cells by preventing mitochondrial dysfunction. Int. J. Mol. Med..

[50] Ginet V., van de Looij Y., Petrenko V., Toulotte A., Kiss J., Hüppi P.S., Sizonenko S.V. (2016). Lactoferrin during lactation reduces lipopolysaccharide-induced brain injury. BioFactors.

[51] Mulder A.M., Connellan P.A., Oliver C.J., Morris C.A., Stevenson L.M. (2008). Bovine lactoferrin supplementation supports immune and antioxidant status in healthy human males. Nutr. Res..

[52] Grey A., Zhu Q., Watson M., Callon K., Cornish J. (2006). Lactoferrin potently inhibits osteoblast apoptosis, via an LRP1-independent pathway. Mol. Cell Endocrinol..

[53] Rascón-Cruz Q., Siqueiros-Cendón T.S., Siañez-Estrada L.I., Villaseñor-Rivera C.M., Ángel-Lerma L.E., Olivas-Espino J.A., León-Flores D.B., Espinoza-Sánchez E.A., Arévalo-Gallegos S., Iglesias-Figueroa B.F. (2024). Antioxidant Potential of Lactoferrin and Its Protective Effect on Health: An Overview. Int. J. Mol. Sci..

[54] Ke D., Wang X., Lin Y., Wei S. (2022). Lactoferrin promotes the autophagy activity during osteoblast formation via BCL2-Beclin1 signaling. Mol. Biol. Rep..

[55] Huang L., Chen R., Liu L., Zhou Y., Chen Z. (2021). Lactoferrin ameliorates pathological cardiac hypertrophy related to mitochondrial quality control in aged mice. Food Funct..

[56] Bradley E., Haran J. (2024). The human gut microbiome and aging. Gut Microbes.

[57] Jackson M.A., Jeffery I.B., Beaumont M., Bell J.T., Clark A.G., Ley R.E., O’toole P.W., Spector T.D., Steves C.J. (2016). Signatures of early frailty in the gut microbiota. Genome Med..

[58] Wilmanski T., Diener C., Rappaport N., Patwardhan S., Wiedrick J., Lapidus J., Earls J.C., Zimmer A., Glusman G., Robinson M. (2021). Gut microbiome pattern reflects healthy ageing and predicts survival in humans. Nat. Metab..

[59] Xie P., Pei Y., Xu L., Shan Y., Cao X. (2026). Short-Chain Fatty Acids: Bridging Gut Microbiota and Systemic Aging—Mechanisms, Interventions, and Current Challenges. Metabolites.

[60] Ashraf M.F., Zubair D., Bashir M.N., Alagawany M., Ahmed S., Shah Q.A., Buzdar J.A., Arain M.A. (2024). Nutraceutical and Health-Promoting Potential of Lactoferrin, an Iron-Binding Protein in Human and Animal: Current Knowledge. Biol. Trace Elem. Res..

[61] Oda H., Wakabayashi H., Yamauchi K., Abe F. (2014). Lactoferrin and bifidobacteria. Biometals.

[62] Mikogami T., Marianne T., Spik G. (1995). Effect of intracellular iron depletion by picolinic acid on expression of the lactoferrin receptor in the human colon carcinoma cell subclone HT29-18-C1. Biochem J..

[63] Hablass F.H., Lashen S.A., Alsayed E.A. (2021). Efficacy of Lactoferrin with Standard Triple Therapy or Sequential Therapy for Helicobacter pylori Eradication: A Randomized Controlled Trial. Turk. J. Gastroenterol..

[64] Fulop T., Larbi A., Hirokawa K., Cohen A.A., Witkowski J.M. (2020). Immunosenescence is both functional/adaptive and dysfunctional/maladaptive. Semin Immunopathol..

[65] Tagliamento M., Frelaut M., Baldini C., Naigeon M., Nencioni A., Chaput N., Besse B. (2022). The use of immunotherapy in older patients with advanced non-small cell lung cancer. Cancer Treat. Rev..

[66] Liu Z., Liang Q., Ren Y., Guo C., Ge X., Wang L., Cheng Q., Luo P., Zhang Y., Han X. (2023). Immunosenescence: Molecular mechanisms and diseases. Signal Transduct. Target. Ther..

[67] Arain M.A., Khaskheli G.B., Barham G.S., Marghazani I.B. (2024). Lactoferrin’s role in modulating NF-κB pathway to alleviate diabetes-associated inflammation: A novel in-silico study. Heliyon.

[68] Oda H., Wakabayashi H., Tanaka M., Yamauchi K., Sugita C., Yoshida H., Abe F., Sonoda T., Kurokawa M. (2021). Effects of lactoferrin on infectious diseases in Japanese summer: A randomized, double-blinded, placebo-controlled trial. J. Microbiol. Immunol. Infect..

[69] Vitetta L., Coulson S., Beck S.L., Gramotnev H., Du S., Lewis S. (2013). The clinical efficacy of a bovine lactoferrin/whey protein Ig-rich fraction (Lf/IgF) for the common cold: A double blind randomized study. Complement Ther. Med..

[70] Kawakami H., Park H., Park S., Kuwata H., Shephard R., Aoyagi Y. (2015). Effects of enteric-coated lactoferrin supplementation on the immune function of elderly individuals: A randomised, double-blind, placebo-controlled trial. Int. Dairy J..

[71] Jing J.L., Ning T.C.Y., Natali F., Eisenhaber F., Alfatah M. (2022). Iron Supplementation Delays Aging and Extends Cellular Lifespan through Potentiation of Mitochondrial Function. Cells.

[72] Zeidan R.S., Han S.M., Leeuwenburgh C., Xiao R. (2021). Iron homeostasis and organismal aging. Ageing Res. Rev..

[73] Mangan D. (2021). Iron: An underrated factor in aging. Aging.

[74] Ru Q., Li Y., Chen L., Wu Y., Min J., Wang F. (2024). Iron homeostasis and ferroptosis in human diseases: Mechanisms and therapeutic prospects. Signal Transduct. Target. Ther..

[75] Patel K.V. (2008). Epidemiology of anemia in older adults. Semin Hematol..

[76] Zeidan R.S., Martenson M., Tamargo J.A., McLaren C., Ezzati A., Lin Y., Yang J.J., Yoon H.-S., McElroy T., Collins J.F. (2024). Iron homeostasis in older adults: Balancing nutritional requirements and health risks. J. Nutr. Health Aging.

[77] Erra Díaz F., Dantas E., Geffner J. (2018). Unravelling the Interplay between Extracellular Acidosis and Immune Cells. Mediat. Inflamm..

[78] Lepanto M.S., Rosa L., Cutone A., Conte M.P., Paesano R., Valenti P. (2018). Efficacy of Lactoferrin Oral Administration in the Treatment of Anemia and Anemia of Inflammation in Pregnant and Non-pregnant Women: An Interventional Study. Front Immunol..

[79] Lönnerdal B., Bryant A. (2006). Absorption of iron from recombinant human lactoferrin in young US women2. Am. J. Clin. Nutr..

[80] Nappi C., Tommaselli G.A., Morra I., Massaro M., Formisano C., DI Carlo C. (2009). Efficacy and tolerability of oral bovine lactoferrin compared to ferrous sulfate in pregnant women with iron deficiency anemia: A prospective controlled randomized study. Acta Obstet. Gynecol. Scand..

[81] Paesano R., Berlutti F., Pietropaoli M., Goolsbee W., Pacifici E., Valenti P. (2010). Lactoferrin efficacy versus ferrous sulfate in curing iron disorders in pregnant and non-pregnant women. Int. J. Immunopathol. Pharmacol..

[82] Paesano R., Pacifici E., Benedetti S., Berlutti F., Frioni A., Polimeni A., Valenti P. (2014). Safety and efficacy of lactoferrin versus ferrous sulphate in curing iron deficiency and iron deficiency anaemia in hereditary thrombophilia pregnant women: An interventional study. Biometals.

[83] Paesano R., Pietropaoli M., Gessani S., Valenti P. (2009). The influence of lactoferrin, orally administered, on systemic iron homeostasis in pregnant women suffering of iron deficiency and iron deficiency anaemia. Biochimie.

[84] Paesano R., Torcia F.T., Berlutti F.B., Pacifici E.P., Ebano V.E., Moscarini M.M., Valenti P.V. (2006). Oral administration of lactoferrin increases hemoglobin and total serum iron in pregnant women. Biochem Cell Biol..

[85] Zhao X., Zhang X., Xu T., Luo J., Luo Y., An P. (2022). Comparative Effects between Oral Lactoferrin and Ferrous Sulfate Supplementation on Iron-Deficiency Anemia: A Comprehensive Review and Meta-Analysis of Clinical Trials. Nutrients.

[86] Guo Y., Guan T., Shafiq K., Yu Q., Jiao X., Na D., Li M., Zhang G., Kong J. (2023). Mitochondrial dysfunction in aging. Ageing Res. Rev..

[87] Somasundaram I., Jain S.M., Blot-Chabaud M., Pathak S., Banerjee A., Rawat S., Sharma N.R., Duttaroy A.K. (2024). Mitochondrial dysfunction and its association with age-related disorders. Front. Physiol..

[88] Sun N., Youle R.J., Finkel T. (2016). The Mitochondrial Basis of Aging. Mol. Cell.

[89] Di Lorenzo R., Marzetti E., Coelho-Junior H.J., Calvani R., Pesce V., Landi F., Leeuwenburgh C., Picca A. (2025). Iron Metabolism and Muscle Aging: Where Ferritinophagy Meets Mitochondrial Quality Control. Cells.

[90] Xu J., Marzetti E., Seo A.Y., Kim J.-S., Prolla T.A., Leeuwenburgh C. (2010). The emerging role of iron dyshomeostasis in the mitochondrial decay of aging. Mech. Ageing Dev..

[91] Stanojevic N., Wohlgemuth S., Zeidan R.S., Lou X., Tamargo J.A., Bravo B., Newman J., Picca A., Marzetti E., Wu K. (2026). Iron dysregulation and mitochondrial dysfunction in aging: A longitudinal study on mobility decline in low- and high-functioning older adults. Exp. Gerontol..

